# Nest survival rate of Reeves's pheasant (*Syrmaticus reevesii*) based on artificial nest experiments

**DOI:** 10.13918/j.issn.2095-8137.2017.008

**Published:** 2017-01-18

**Authors:** Xu Luo, Yu-Ze Zhao, Jing Ma, Jian-Qiang Li, Ji-Liang Xu

**Affiliations:** School of Nature Conservation, Beijing Forestry University, Beijing 100083, China; School of Nature Conservation, Beijing Forestry University, Beijing 100083, China; School of Nature Conservation, Beijing Forestry University, Beijing 100083, China; School of Nature Conservation, Beijing Forestry University, Beijing 100083, China; School of Nature Conservation, Beijing Forestry University, Beijing 100083, China

**Keywords:** Reeves's pheasant, *Syrmaticus reevesii*, Nest survival rate, Artificial nest experiments

## Abstract

To explore the nest survival rate of Reeves's pheasant (*Syrmaticus reevesii*) and the nest-site factors that affect it, we conducted artificial nest experiments with reference to natural nests at Dongzhai National Nature Reserve (DNNR), Henan Province and Pingjingguan, Hubei Province from April to June 2014 simulating the situation in its early and later breeding season. We also determined distance characteristics of the nest sites using ArcGIS 10.0. Nest survival models were constructed in Program MARK for data analysis. Results indicated that in the early breeding season, the apparent survival rate (ASR) in DNNR (52.4%) was significantly greater than that in Pingjingguan (13.5%), and the ASR in the later breeding season in DNNR (26.7%) was not indistinctively correlated with Pingjingguan (3.2%). The daily survival rate (DSR) in the later breeding season was 93.8% in DNNR and 92.0% in Pingjingguan, respectively. The DSRs were both negatively correlated with nest distance to forest edges and settlements. The DSR in Pingjingguan was positively correlated with nest distance to paths and negatively correlated with nest distance to water sources. However, the DSR in DNNR was negatively correlated with nest distance to paths but positively correlated with nest distance to water sources.

## INTRODUCTION

Reeves's pheasant (*Syrmaticus reevesii*) is a rare and endangered endemic species in China. It is listed as Grade Ⅱ wildlife under national protection (State Council, 1988) and as a vulnerable species by the International Union for the Conservation of Nature and Natural Resources (IUCN, 2015). Due to habitat destruction (Wu & Xu, 1987), poaching (Xu et al., 1996) and predation pressure from natural enemies (Xu et al., 1996), the Reeves's pheasant population is decreasing at a rate of 20% every ten years (Zhou et al., 2015). The threats to Reeves's pheasant survival are considered more severe than those of many Grade I protected species in China (Zhang et al., 2003; Zhou et al., 2015). The Reeves's pheasant is ground nesting and highly vigilant, which makes it difficult to carry out field research and nest tracking (Lei & Lu, 2006; Zhang et al., 2004). Direct observation of incubating females can induce nest abandonment and breeding failure (Collar et al., 1994), and thus only limited information is known in regards to their nest survival rate.

Nest building, egg laying and egg hatching are not only critical stages in bird breeding seasons, but are also relatively precarious and easily affected by adverse environmental factors (Welty, 1962). Nest success rate is one of the most important factors determining fertility and has significant effects on bird population quantities (Beale & Monaghan, 2004). Accurate estimates of nest success rate and relevant influencing factors are important to understand the quantitative dynamics of bird populations and meaningful to enact suitable protective policies for endangered species (Lindell et al., 2011). 

Artificial nest experiments using quail (*Coturnix japonica*), chicken, or artificial plaster eggs to similar to natural nests have been used to investigate predation risks of nestling (Reitsma et al., 1990; Sun et al., 2011). In addition, because it is easy to manipulate and permitting to control the experimental conditions, artificial nest experiments have also been popular in the studies on nest survival rate (Major & Kendal, 1996; Martin, 1987; Sieving, 1992; Sun et al., 2011; Willebrand & MarcstrÖm, 1988), and are a valuable reference for natural nesting survival studies (Wilson et al., 1998). Wang et al. (2016) used artificial nest experiments to study predation of the Reeves's pheasant. Results indicated that, to a certain extent, artificial nest experiments can reflect the fates of natural nests. Referencing the methods used in Wang et al. (2016), we conducted artificial nest experiments on Reeves's pheasant at Dongzhai National Nature Reserve (DNNR), Henan Province and Pingjingguan, Suizhou City, Hubei Province from April to June 2014. Experiments were based on the characteristics of natural nests collected from field surveys in recent years, with chicken eggs used as mock eggs. We aimed to provide evidence on the nest survival rates of Reeves's pheasant, and the effects of distances from nest sites to various habitat parameters (e.g., forest edges, paths, water sources, settlements) on survival rates.

## MATERIALS AND METHODS

### Study area

The experiments were carried out at Baiyun Protection Station, DNNR, Henan Province (N31°28′-32°09′, E114°18′-114°30′) and Pingjingguan, Suizhou City, Hubei Province (N31°51′-31°52′, E113°54′-113°55′). The two areas are in the south foothills of Dabieshan Mountain, less than 40 km apart and both approximately 400 hm^2^ in area. The geological conditions, precipitation, temperature and phenology are similar (details in Wang et al., 2016).

### Locating natural nests

Natural Reeves's pheasant nests in Pingjingguan were located in two ways: (1) Fouteen nests were found by radio telemetry during field surveys from 2011 to 2013, seven nests were found during this study from ten captured females caught in March 2013; and, (2) Four nests were found by interviewing local residents undertaking farming and firewood, herb collection in the mountains. The natural nests included in DNNR were seven nests found by other research teams during 2011 to 2014.

Information on the structures and sites of the fourteen natural nests obtained from Pingjingguan during 2011 to 2013 were used as references for establishing artificial nests. The twenty-five natural nests from Pingjingguan and seven from DNNR during 2011 to 2014 were included in evaluating and comparing the survival rates between natural and artificial nests.

### Artificial nest experiments

The locations of the artificial nests were chosen within the areas in which Reeves's pheasants were found by radio telemetry during previous research (Sun et al., 2003; Xu et al., 2007; Bai, 2013). The selection of the artificial nests was determined based on the habitat characteristics of fourteen natural nests found during field surveys from 2011 to 2013. The artificial nests were constructed by mimicking the structures of natural nests (Wang et al., 2016). The artificial nests were disk-like, padded with withered and yellow* Castanea mollissima* leaves, *Pinus tabuliformis* pine needles or straw, and covered with 1-2 down feathers collected from the field. Four chicken eggs of similar size and color to that of Reeves's pheasant were placed in each artificial nest.

Two rounds of artificial nest experiments were conducted. The first round was carried out from late April. When choosing locations by ArcGIS 10.0(Esri Inc. <*http://www.esri.com/*>), seventy random locations were picked in the two study areas. Except for the locations with rocks, roads, nudations, and ponds, one artificial nest locus was picked within a 100 m^2^ range around each of the remaining locations. A total of 46 and 42 artificial nests were placed at Pingjingguan and DNNR, respectively. Using the same method and procedure in the second round of experiments (May 2014), fifty random locations were determined, and a total of 31 and 30 artificial nests were placed at Pingjingguan and DNNR, respectively. As per the descriptions of the four stages of breeding season in Reeves's pheasant (Sun et al., 2003), our two experiments simulated nest survival situations in the early and later stages, respectively.

In natural environments, the hatching period of Reeves's pheasant is 26-27 d (Zhang et al., 2004). In this study, the period of the artificial nest experiments was set at 30 d. Due to adverse weather conditions during the first experiment, the nests were checked three times on day 10, day 15 and day 30. During the second experiment, the nests were checked every five days The inspecting time was defined as interval of checked and setting up day (Dinsmore et al., 2002).

To reduce researcher influence on the experiments, human disturbance was minimized during nest checking, e.g., leaving few footprints around the artificial nest and avoiding behaviors that might affect nest, such as touching the eggs (Driscoll et al., 2005). In the wild, if a nest is disturbed or eggs in the nest are preyed, the female Reeves's pheasant abandon the nests (Johnsgard, 1999). If eggs inside the artificial nest were destroyed, removed or disappeared, the eggs were not replaced (Nour et al., 1993) and the nest was defined as failed (Noske et al., 2008). Otherwise, the nest was considered one survival nest. Moreover, because Reeves's pheasant is precocial, one success nest was defined if it was a survival nest at the last inspecting time.

### Distance parameters of nest locations

The GPS locations of both natural and artificial nests were analyzed by ArcGIS 10.0. By referencing the definitions of habitat parameters by Xu et al. (2006) and Wang et al. (2016), the degree of slope (Sld), distance to water resources (Dwt), paths (Dho), forest edges (Dfb) and settlements (Dro) were determined. The degree of slope was calculated by Slope tool and the minimum distance parameters were obtained by Euclidean Distance tool.

### Data analysis

To compare our findings with previous reports, the apparent survival rate, i.e. ASR was defined in accordance with the definitions of Driscoll et al. (2005):

1 ${\rm{ASR}} = {n_{\rm{s}}}/\left( {{n_{\rm{s}}} + {n_{\rm{f}}}} \right)$

Where, ASR is the apparent survival rate, *n*_s_ represents the number of successful nests, and *n*_f_ represents the number of failed nests.

*χ*^2^-tests were used to compare the differences in ASRs between artificial and natural nests, as well as the differences in ASRs between the two study areas and two different study periods. The results were examined by SPSS 22.0 and plotting was conducted by Sigma Plot 12.1.

Daily survival rates, i.e. DSRs were calculated by Program MARK (V8.0). Due to the irregular checking intervals in the first experiment, only data from the second experiment were included in calculating the DSR. In the calculation, five basic variables were used for the nest survival rate obtained by the module Nest Survival within MARK program: (1) setting up day of artificial nests (all in "1"); (2) last survival day of nest; (3) the day of nest fate (failure or success) determined; (4) the fate of nest (failure or success); and (5) quantity of nests with same fate. None of the variables went through standardization. Selecting sin option in Link Function to construct the stable model of the DSR of the artificial nests in the later breeding season, and the DSRs of the two study areas in the later breeding season were calculated.

To clarify if degree of slope and distance parameters of nest location to various habitat factors affected DSR, five variables, Sld, Dho, Dfb, Dwt and Dro, were added into the model Nest Survival. By combining the five previously described basic variables, a model regarding the correlations among DSR, degree of slope and distance parameters was constructed. The model average estimated value (*θ*±*SE*) of each variable and 95% confidence interval, i.e. 95% CI were obtained. The plus-minus estimated value excluding "0" indicated that this variable had positive and negative effects on model construction.

In the results, data of successional variables were presented as mean±*SE*, where, mean is the arithmetical mean and *SE* is the standard deviation. A *P*-value of <0.05 represented significant differences.

## RESULTS

### Apparent survival rates between artificial and natural nests

**Figure 1:**
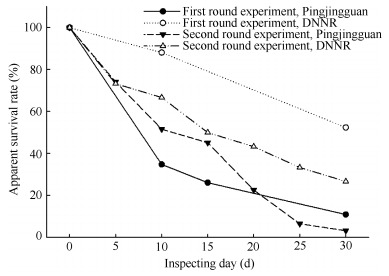
Apparent survival rates over the two experimental periods in the two study areas

The ASRs of the artificial nests in the two study areas decreased with time (Figure 1). At the end of the experiments, the ASRs of artificial nests in Pingjingguan and DNNR were 11.6% (*n*=9) and 41.7% (*n*=30), respectively; that of the twenty-five natural nests in Pingjingguan was 20.0%, and that of the seven natural nests in DNNR was 28.5%. The χ^2^-test results showed no significant differences in the ASRs between artificial and natural nests during the two experiments (Pingjingguan: *χ*^2^=0.67, *df*=1, *P*=0.41; DNNR: *χ*^2^=0.20, *df*=1, *P*=0.65).

### Apparent survival rates of artificial nests at the two study areas in different time periods

At the end of the early breeding season, the ASR of the artificial nests in Pingjingguan was 13.5% (*n*=6), significantly lower than that in DNNR (52.4%, *n*=22) (*χ*^2^=7.65, *df*=1, *P*<0.01). At the end of the later breeding season, the ASR of artificial nests in Pingjingguan was 3.2% (*n*=1), also lower than that of DNNR (26.7%, *n*=8), though the difference was not significant (*χ*^2^=3.81, *df*=1, *P*>0.05). In the two study areas, the ASRs of artificial nests during later breeding season were both lower than that during the early breeding season. However, significant differences were only found in DNNR (Pingjingguan: *χ*^2^=4.75, *df*=1, *P*>0.05; DNNR: *χ*^2^=5.12, *df*=1, *P*<0.05).

### Daily survival rate during the later breeding season and its variations with distance and degree of slope

In the later breeding season, the stable model results showed that the DSRs in DNNR and Pingjingguan were 93.8% (*n*=30, CI_95_=90.8%-95.9%) and 92.0% (*n*=31. CI_95_= 88.7%-94.3%), respectively.

**Figure 2:**
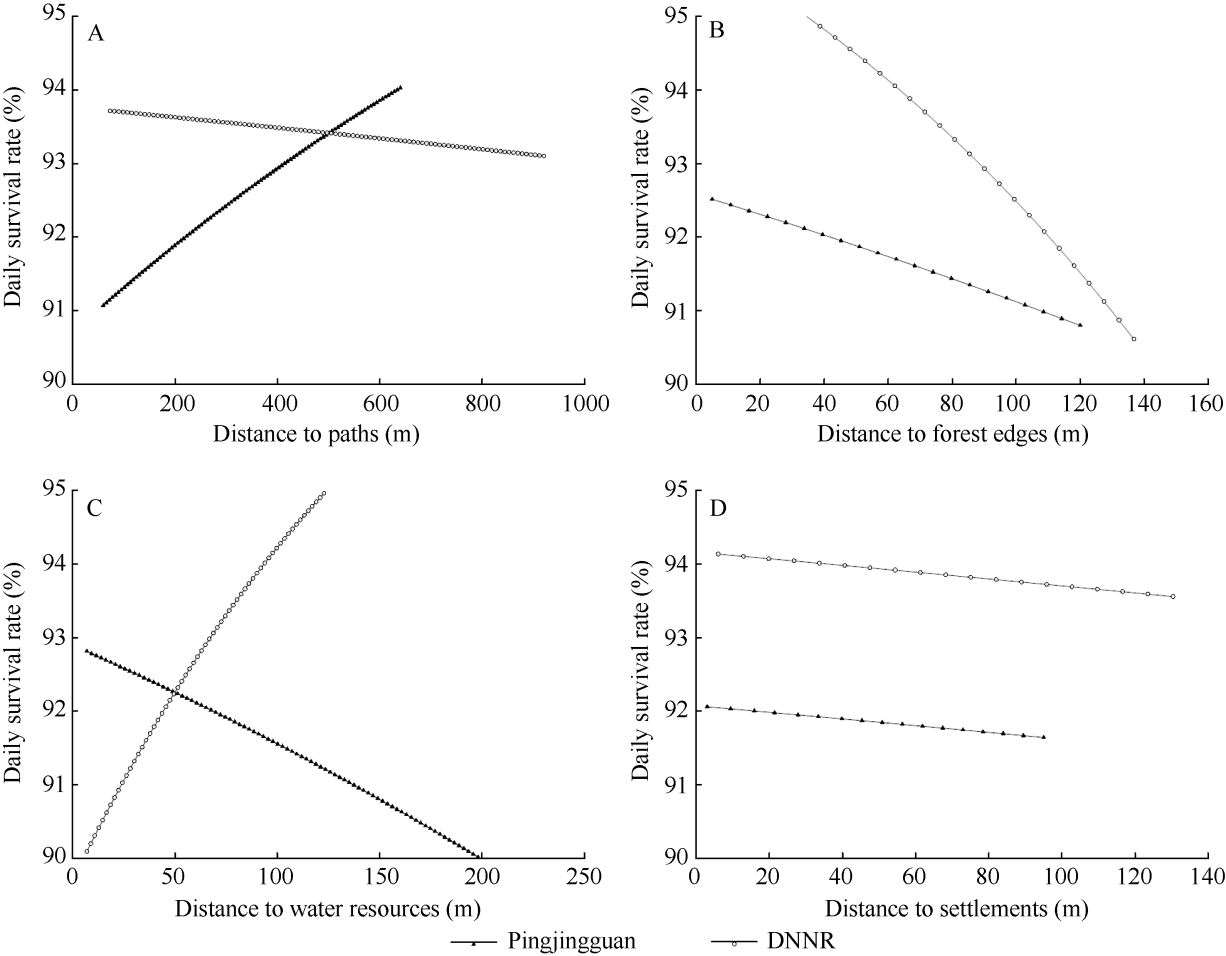
Daily survival rates of artificial nests in the later breeding season with different nest distances to habitat parameters

The distance parameter analyses of model Nest Survival showed that in the later breeding season, DSRs of artificial nests in the two study areas were negatively correlated with distances to forest edges and settlements (Figure 2). In DNNR, the DSR decreased considerably with increasing distance from nest location to forest edges. Moreover, different correlation patterns of DSRs with distance to paths and water resources were found in DNNR and Pingjingguan (Figure 2). In Pingjingguan, the DSR was positively correlated with distance to paths, but negatively correlated with distance to water resources. However, these parameters effects showed the opposite pattern in DNNR.

## DISCUSSION

Reeves's pheasants are highly vigilant birds, which makes it difficult to track their nests in the field and carry out monitoring (Lei & Lu, 2006; Zhang et al., 2004). Artificial nest experiments can decrease human disturbance to hatching in the field and provide sufficient samples for research purposes (Major & Kendal, 1996; Sieving, 1992; Sun et al., 2011; Wang et al., 2016; Wilson et al., 1998). In this study, the ASRs of artificial nests during the two experiments were comparable with that of natural nests, indicating their ability to mimic, to some extent, the survival situation of Reeves's pheasant nests in the field.

In the two artificial nest experiments, the ASRs during the later breeding season were both lower than those during the early breeding season. This phenomenon that nest survival rates vary or decrease with time has been reported in previous research (Daan et al., 1990). Our results also support this finding, which could relate to the increase in predation pressure and human disturbance with time (Hatchwell, 1991; Becker & Zhang, 2011).

In the two study areas, during the artificial nest experiments in late May (the later breeding season), the number of raptors, such as *Butastur indicus* and *Accipiter soloensis*, increased, and as they also began to breed, predation pressure to the Reeves's pheasant increased as well. Therefore, predation threats to artificial nests were more severe than that during the early breeding season. According to the local DNNR survey conducted by Ma et al. (2012), because the later breeding season of Reeves's pheasant overlaps with the peak period of busy farming, e.g., pasturing, chestnut weeding, human disturbance in the later breeding season was more intense than that in the early breeding season. In Ma's survey to the community, 35.3% of interviewees claimed to collect Reeves's pheasant eggs from April to June. Sun et al. (2011) stated that repetitive artificial nest experiments in one breeding season could allow predators to adapt to human traces, with increases in predation risks in later experiments.

In this study, in the later breeding season, the DSRs in the two study areas both decreased with increasing distance from nests to the forest edges. The edge effect hypothesis indicates that the edging areas of habitats usually have more enriched vegetation resources and more complicated environments than central areas, so predation pressure at edging areas is higher (Ewers & Didham, 2007; Fahrig, 2003). Our results are discordant with the edge effect hypothesis. It is possible that because human activities are more intense in edging areas, predators, especially those that mammalian predators, are forced into the less disturbed central areas. Therefore, predation pressures in central areas are higher than that in edging areas. Moreover, because it is more common for a nest to be destroyed by a predator than by a human, the effect of predation pressure on nest survival is higher than that of human disturbance. Wang et al. (2016) found that in the later breeding season, the major predation pressure came from predators preying on animals, which supports our assumption to a certain extent. Moreover, the negative correlation between DSR and nest distance to settlements in both study areas also suggests that human disturbance relieved potential predation pressures of artificial nests.

We also found that in the later breeding season, with increasing distance from the nests to paths, the DSR of artificial nests in Pingjingguan increased, whereas that in DNNR showed the opposite trend. The possible reasons for this are that the paths in Pingjingguan are also the main roads used by residents entering the mountain area; therefore, compared with DNNR, the habitats near paths in Pingjingguan have smaller vegetation coverage, fewer herbaceous plants, and higher human disturbance. Therefore, the nests closer to the paths were under higher predation pressure and human disturbance. Moreover, the distance of nests to water resources also had different effects on DSRs in the two study areas. In DNNR, the DSR increased with increasing distance to water resources. The reason might be that predators are more active near water resources, and thus threats from natural enemies decreased with distance to water resources and the artificial nests closer to water resources were under higher predation pressure. However, the situation in Pingjingguan exhibited the opposite pattern, and it was assumed that because water resources, e.g., river, penstock, were in areas with more frequent farming activities, disturbance from livestock and herdsman suppressed activities of predators nearby.

In summary, artificial nest experiments were conducted to mimic the survival rates of Reeves's pheasant's nests. Results showed low nest survival rates and high impacts of nest location. Our findings indicate that although data obtained from natural nests allow for more persuasive assumptions (Paton, 1994), to vigilant and endangered species, such as Reeves's pheasant, using artificial nests to mimic natural nests is a useful method in research.

## ACKNOWLEDGEMENTS

We thank Qin-Yun Wang, Peng Zhao, and Jun-Qin Hua for help in collecting data in the fields; residents Yuan-Jun Zhang and Jin-Dong Chen for their help; and staff from the Administration Department of Dongzhai National Nature Reserve and Pingjingguan village committee for supporting this study.
